# Engineered Extracellular Vesicles for Intervertebral Disc Regeneration: Mechanisms, Strategies, and Translational Potential

**DOI:** 10.7759/cureus.97119

**Published:** 2025-11-17

**Authors:** Jiliang Wang, Bing Ran, Jun Wei

**Affiliations:** 1 Department of Pain, Gannan Medical University, Ganzhou, CHN; 2 Department of Pain, The First Affiliated Hospital of Gannan Medical University, Ganzhou, CHN

**Keywords:** biomaterials based scaffolds, extracellular vesicles, intervertebral disc degeneration (ivdd), tissue engineering and regenerative medicine, translational potential

## Abstract

Intervertebral disc degeneration (IVDD) is a primary contributor to chronic low back pain and disability, yet current treatment strategies remain largely palliative, with limited regenerative potential. Engineered extracellular vesicles (EVs) have emerged as a promising class of bio-nanocarriers, offering low immunogenicity, high biocompatibility, and precise therapeutic cargo delivery. This review systematically summarizes recent advancements in EV engineering approaches, including cargo loading techniques, surface functionalization, and integration with stimuli-responsive biomaterials, and delineates their mechanistic roles in modulating inflammation, cellular senescence, and extracellular matrix remodeling in the degenerated disc microenvironment. Furthermore, we structure the discussion around a “carrier-mechanism-application” axis, providing a conceptual and technical framework to guide the development of EV-based regenerative therapies. Finally, we address current translational challenges and propose future directions to bridge the gap between bench and bedside in the context of IVDD treatment.

## Introduction and background

Intervertebral disc degeneration (IVDD) is a major cause of chronic low back pain, which affects a significant proportion of the global population and imposes substantial socioeconomic burdens. The intervertebral disc is composed of the following three main structures: the nucleus pulposus (NP), annulus fibrosus (AF), and cartilage endplate (CEP). Among these, degeneration of the NP, characterized by extracellular matrix (ECM) breakdown, loss of proteoglycans, and abnormal collagen remodeling, is considered a central event driving the overall degenerative process. These structural and biochemical alterations impair the disc’s capacity to maintain hydration and mechanical resilience, ultimately contributing to disc height loss and inflammation within the spinal microenvironment [1].

**Figure 1:**
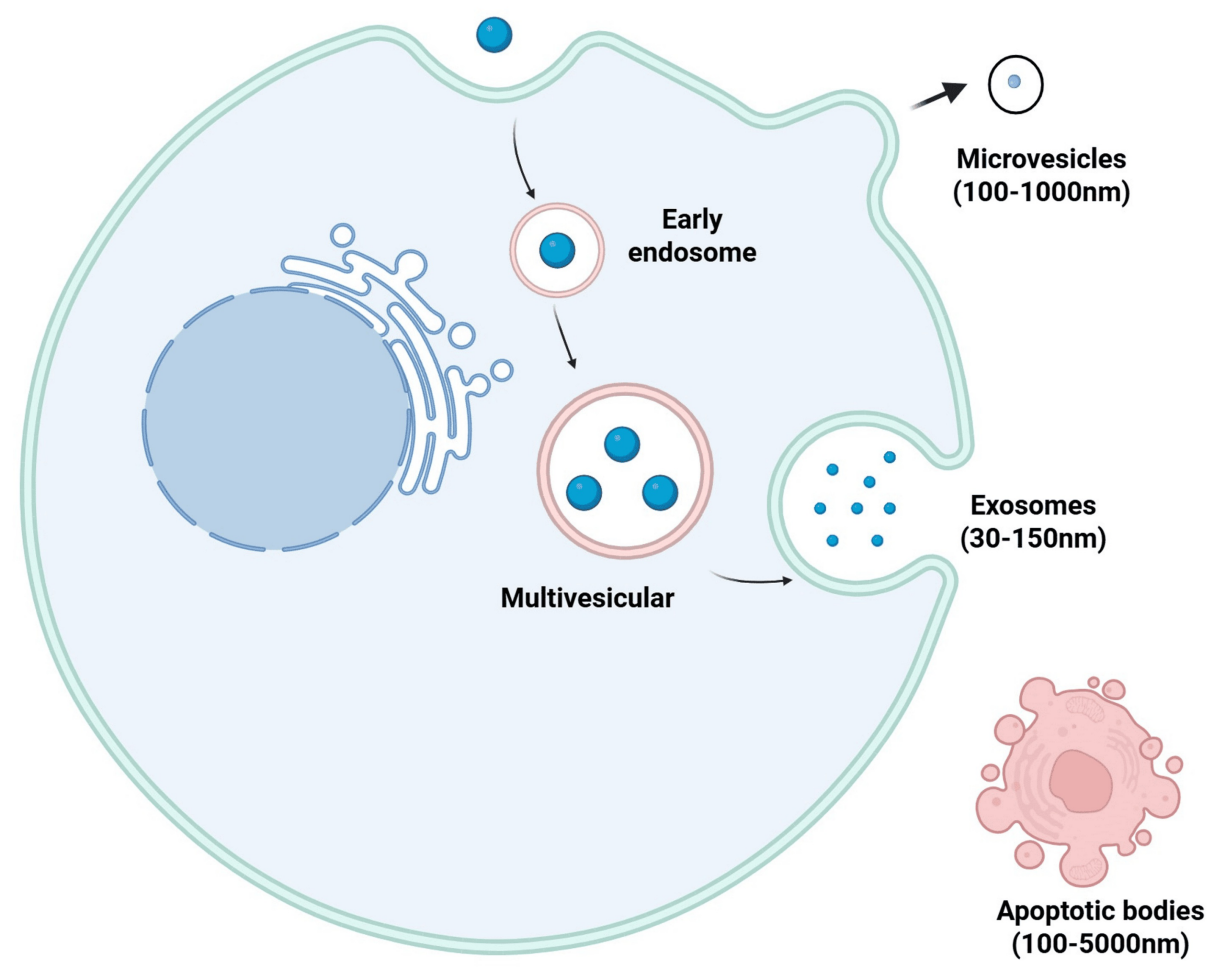
Secretion of extracellular vesicles. This image is created by the authors of this study using BioRender.com (agreement number: KF28VQM6RD).

In recent years, extracellular vesicles (EVs) have emerged as critical mediators of intercellular communication in both physiological and pathological contexts. EVs are non-replicative nanoparticles enclosed by a lipid bilayer and are widely distributed in body fluids (such as plasma, saliva, and urine) and tissues [2,3]. The essential role of EVs is to facilitate intercellular communication via the delivery of biologically active molecules, including proteins, nucleic acids (mRNA/miRNA), and lipids [4]. Based on their origin and biogenesis, EVs are primarily categorized into exosomes (30-150 nm), microvesicles (100-1000 nm), and apoptotic bodies (100-5000 nm), each exhibiting distinct characteristics and biological functions [5]. The formation of exosomes depends on the fusion of multivesicular bodies (MVBs) with the plasma membrane. MVBs, which express lysosome-associated membrane proteins such as LAMP1 and LAMP2, partially fuse with the cell membrane to facilitate exosome release [6,7]. In contrast, microvesicles are released via outward budding of the plasma membrane, a process regulated by calcium influx, whereas apoptotic bodies are formed through programmed cell death, encompassing components of the parent cell and participating in immune regulation [8,9]. Together, these subtypes of EVs serve as pivotal mediators of physiological and pathological processes (Figure 1).

Nucleus pulposus (NP) degeneration, a key pathological process in intervertebral disc degeneration, is closely linked to an imbalance in extracellular matrix (ECM) homeostasis. Loss of proteoglycans and abnormal collagen cross-linking in the NP lead to reduced osmotic pressure and impaired mechanical function [10]. This structural deterioration further compromises the disc’s ability to absorb compressive loads, accelerating degenerative changes. Single-cell transcriptomics reveals 11 distinct cell subpopulations in degenerated NP tissue, comprising six NP subtypes (homeostatic, adhesive, inflammatory, ER stress-responsive, fibrocartilage-like, and progenitor NP cells) and five immune cell types, including macrophages and T cells. During the progression of IVDD, metabolic homeostatic NP cells are most abundant in early-stage (Pfirrmann grade II) degeneration, while fibrocartilage-like NP cells become more prominent in the intermediate (grade III) and advanced (grade IV) stages. This cellular transition reflects a shift from a reparative to a fibrotic phenotype, indicating a worsening microenvironment. Simultaneously, there is a notable decline in M2 macrophages and a progressive rise in M1 macrophages, amplifying the inflammatory response cascade [11]. Given the complexity of cellular and immune responses in NP degeneration, targeted molecular modulators such as EVs are emerging as promising therapeutic tools. EVs can attenuate degeneration by delivering miRNAs that target RASSF5, thereby inhibiting the release of inflammatory cytokines such as IL-6 and IL-1β, while also downregulating the expression of genes including SUV39H1, Bax, MMP13, and p62, which suppresses apoptosis in NP tissue [12]. These findings highlight the potential of EV-based interventions to modulate both immune responses and cell survival pathways in degenerative discs.

## Review

Methodology

This review was conducted in accordance with the general principles of the Preferred Reporting Items for Systematic Reviews and Meta-Analyses (PRISMA) framework. A comprehensive literature search was performed exclusively in the PubMed database to ensure the inclusion of high-quality biomedical studies relevant to extracellular vesicle engineering and intervertebral disc regeneration.

Search Strategy

A comprehensive electronic search was performed in the PubMed database to identify relevant studies on extracellular vesicle engineering and intervertebral disc regeneration. The search covered publications from January 2015 to September 2025 and was last updated on September 30, 2025. Both Medical Subject Headings (MeSH) and the following free-text keywords were used, combined with Boolean operators: (“extracellular vesicle” OR “exosome”) AND (“engineering” OR “bioengineering”) AND (“intervertebral disc” OR “disc degeneration” OR “nucleus pulposus” OR “annulus fibrosus” OR “regeneration”). The reference lists of key articles were manually screened to capture additional relevant publications. Only peer-reviewed, English-language original research articles were considered; review papers, commentaries, conference abstracts, and non-English publications were excluded.

Inclusion and Exclusion Criteria

Studies were considered eligible for inclusion if they investigated the engineering or therapeutic application of extracellular vesicles (EVs) or exosomes, reported data directly related to intervertebral disc degeneration or regeneration, and presented original experimental or clinical findings published in English. Articles were excluded if they lacked a clear focus on EV engineering or intervertebral disc biology, if they were classified as review papers, editorials, or conference abstracts, or if they did not provide original research data suitable for qualitative synthesis.

Screening Process

**Figure 2:**
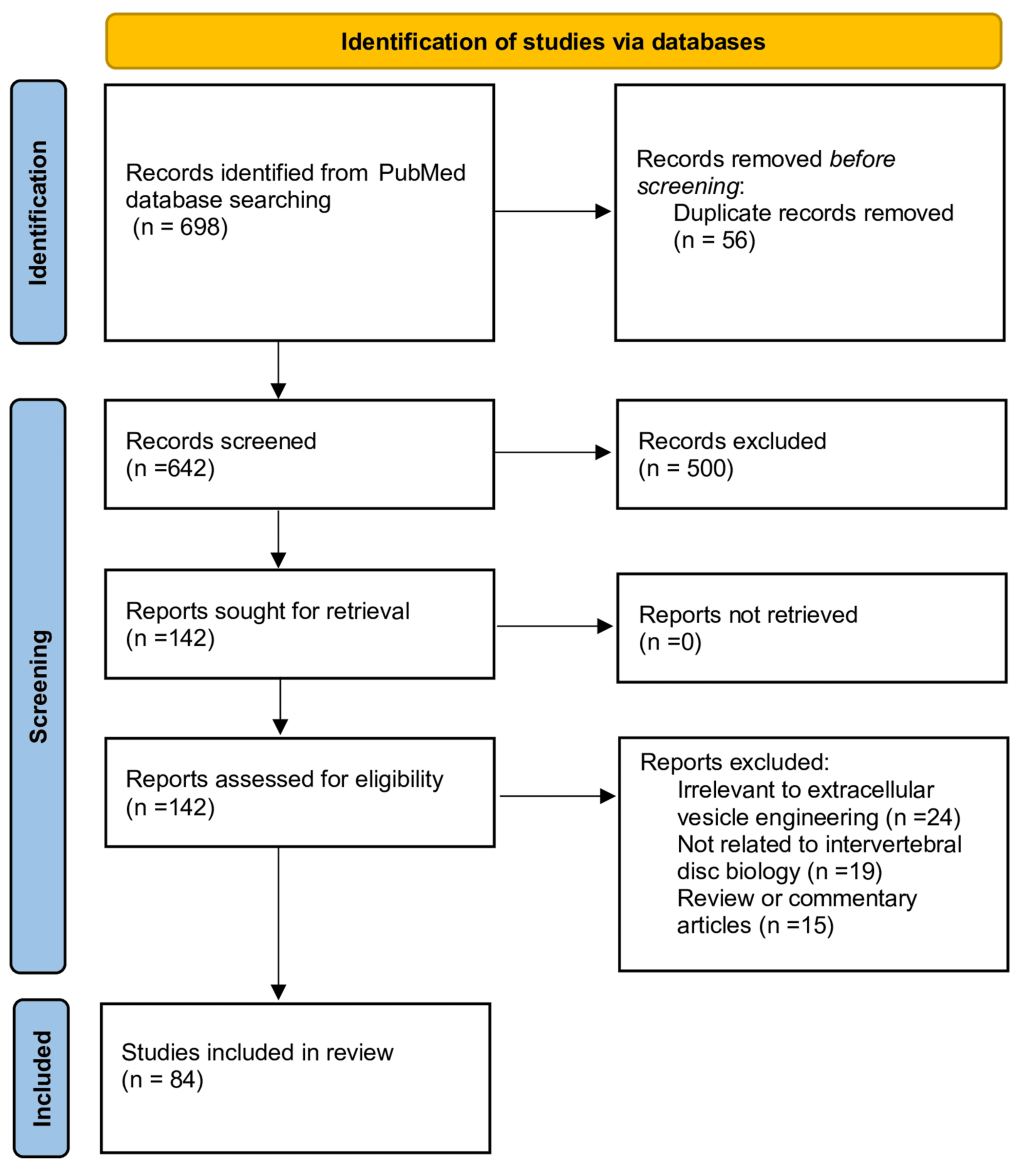
PRISMA flow diagram. PRISMA: Preferred Reporting Items for Systematic Reviews and Meta-Analyses

A total of 698 records were initially retrieved. After removing 56 duplicates, 642 records remained for title and abstract screening. Of these, 500 were excluded because they were irrelevant to the topic or did not present original research data, leaving 142 articles for full-text review. Upon detailed eligibility assessment, 58 studies were excluded due to irrelevance to extracellular vesicle engineering (n=24), lack of relation to intervertebral disc biology (n=19), or classification as review or commentary articles (n=15). Ultimately, 84 studies met the inclusion criteria and were incorporated into the qualitative synthesis. Although the search primarily covered literature published after 2015, one seminal study from 1998 was also included for historical and conceptual completeness, as it represents an early foundational contribution to extracellular vesicle research. Due to heterogeneity among study designs, a meta-analysis was not performed, and results are presented narratively. The study selection process is summarized in the PRISMA flow diagram (Figure 2) [13].

Pathological mechanisms in IVDD and engineering targets

The pathological mechanism of nucleus pulposus degeneration exhibits the following multidimensional cascade: oxidative stress, proliferation inhibition, and pathological apoptosis collectively drive matrix degradation, while aberrant angiogenesis further disrupts the disc microenvironment. These intertwined pathological events exacerbate tissue breakdown and promote a hostile cellular niche. As degeneration progresses (Pfirrmann grade III-IV), the annulus fibrosus loses collagen fiber orientation, the area of endplate calcification expands, ultimately resulting in reduced disc height and biomechanical failure [14]. The following sections elaborate on the major pathological mechanisms and their corresponding engineering intervention targets, including modulation of inflammatory responses, attenuation of oxidative stress, regulation of extracellular matrix remodeling, and intervention in neurovascular ingrowth.

Modulation of Inflammatory Responses

Inflammation is one of the core driving factors of IVDD. Mesenchymal stem cell-derived exosomes (MSC-exos) exert anti-inflammatory effects by regulating macrophage polarization and inflammasome activity [15]. Specifically, evidence indicates that miR-532-5p transported by MSC-exos targets RASSF5, effectively inhibiting TNF-α-induced apoptosis of nucleus pulposus cells, fibrotic matrix accumulation, and ECM degradation [16]. In hyperglycemic environments, EVs from M1 macrophages can reprogram recipient macrophages toward the M2 phenotype, thus reducing inflammation and enhancing tissue regeneration [17]. Furthermore, platelet-rich plasma-derived exosomes (PRP-exos) markedly suppress caspase-1 activation and IL-1β secretion through ubiquitin-mediated degradation of NLRP3, and enhance M2 macrophage infiltration by stimulating STAT6 phosphorylation [18]. These immunomodulatory effects underscore the versatility of EVs in restoring immune balance within the degenerative disc niche. From a bioengineering perspective, human embryonic stem cell-derived exosomes (hESC-exos) deliver miR-302c to target the NLRP3 inflammasome, thereby blocking pyroptotic signaling pathways [19], whereas PRP-derived EVs carrying the lncRNA MALAT1 inhibit NF-κB signaling through the miR-217/SIRT1 pathway [20].

Attenuation of Oxidative Stress

Oxidative stress refers to an imbalance between oxidants and antioxidants within cells, leading to excessive production of reactive oxygen species (ROS). Oxidative stress can exacerbate IVDD via mitochondrial electron transport chain (ETC) leakage and ferroptosis pathways; superoxide production increases under hyperglycemic conditions, whereas MTF1-mediated inhibition of iron transport leads to ferroptosis in nucleus pulposus cells (NPCs) [21,22]. This redox imbalance disrupts mitochondrial integrity and promotes cell death, accelerating disc degeneration. In response, MSC-exos can significantly reduce ROS levels in NPCs, alleviate oxidative injury by restoring mitochondrial dynamics, and reduce NPC apoptosis [23]. They also activate the SIRT1-PGC1α-TFAM axis to sustain mitochondrial function and attenuate IVDD progression through reduction of oxidative stress and inflammatory responses [24]. Notably, an engineered microneedle-delivered polydopamine-exosome system (PDA@Exo MN) achieves a triple-synergistic effect by activating the PI3K/Akt/mTOR signaling axis as follows: significantly scavenging reactive oxygen species, enhancing permeability to improve local drug delivery, and simultaneously promoting osteogenesis while balancing the anabolic and catabolic processes in cartilage [25]. Another type of hydrogel microsphere containing SOD3-enriched exosomes (s-exos) was shown to reduce ROS levels in chondrocytes by 142.7%, promote the synthesis of COL II and ACAN, and maintain the stability of the extracellular matrix [26]. Engineering strategies also include upregulation of miR-141-3p in PRP-exos, which inhibits H2O2-induced pro-inflammatory cytokines and pyroptosis in NPCs [27], whereas a contrasting mechanism involves downregulation of miR-31-5p in MSC-exos, which enhances ATF6-mediated endoplasmic reticulum stress, thereby aggravating apoptosis and calcification of endplate chondrocytes [28]. These findings underscore the importance of fine-tuning EV cargo composition to achieve therapeutic redox modulation.

Regulation of Extracellular Matrix (ECM) Remodeling

ECM metabolic imbalance is characterized by suppressed anabolic activity and elevated catabolic activity, notably increased matrix metalloproteinase 13 (MMP13) activity and reduced type II collagen. Inhibiting the expression of lipocalin 2 (LCN2) significantly enhances type II collagen synthesis and reduces MMP-13 production; this bidirectional regulation effectively maintains ECM homeostasis in both in vitro and animal models [29]. In this context, MSC-exos promote the synthesis of sulfated glycosaminoglycans (s-GAGs), suppress IL-1β-induced nitric oxide and MMP13 production, relieve pain, mitigate inflammation, and facilitate joint restoration [30]. Furthermore, miR-214-3p overexpression upregulates COL2A1 and SOX9, downregulates MMP3 and MMP13, and counteracts cartilage matrix degradation and apoptosis triggered by IL-1β in chondrocytes [31]. To enhance ECM repair in vivo, a decellularized extracellular matrix hydrogel, intricately cross-linked with copper ions through ligand bonds and loaded with FGF2-containing exosomes (exoFGF2@ECM/Cu²⁺ hydrogel), has been shown to promote angiogenesis and collagen deposition [32]. In another EV-based strategy, Zhang et al. used a lipid insertion method to decorate the surface of EVs with cartilage affinity peptide (CAP) and loaded siRNA targeting MMP13 (siMMP13) inside them, thereby generating CAP-Exo/siMMP13. This engineered construct achieves targeted delivery of siRNA to chondrocytes and specific silencing of MMP13, effectively alleviating cartilage degeneration [33]. Collectively, these approaches highlight the capacity of engineered EVs to orchestrate ECM regeneration through multifactorial regulation.

Intervention in Neurovascular Ingrowth

Pathological angiogenesis and neural sensitization are key drivers of IVDD-related pain. Circulating soluble fms-like tyrosine kinase-1 (sFlt-1) induces widespread endothelial dysfunction by binding to and competitively inhibiting vascular endothelial growth factor (VEGF) and placental growth factor (PlGF); sFlt-1-loaded exosomes (sFlt-1-Exo) markedly suppress cell proliferation, migration, and tube formation [34]. In another example of immunomodulatory application, MSC-exosomes target IL-6R via miR-204, reprogramming M1 macrophages into the M2 phenotype, thereby inhibiting corneal epithelial degeneration, increasing central corneal and epithelial thickness, restoring tissue structure, and alleviating dry eye symptoms, such as stinging and redness [35]. Moreover, exosomes loaded with super-repressor IκB (Exo-srIκB) significantly attenuate proinflammatory cytokines, including endoglycan, MPO, osteopontin, pentraxin-3, and serotonin E1/PAI-1, and relieve mechanical hypersensitivity in chronic post-ischemia pain (CPIP) mice by blocking NFκB nuclear translocation [36]. To facilitate localized neural repair, Schwann cell-derived exosomes (SC-exos) encapsulated in a nerve tissue engineering hydrogel constitute a slow-release system that significantly enhances bone regeneration by promoting innervation, immune regulation, angiogenesis, and osteogenesis in vivo. Its mechanism also involves upregulation of the TGF-β1/SMAD2/3 signaling pathway, thereby promoting the osteogenesis of BMSCs [37]. In addition, a biological scaffold based on autologous plasma exosomes (AP-exos), loaded with a neuron-targeting peptide (RVG) and growth-promoting peptides (ILP and ISP), enables targeted delivery to neurons at the injury site, triggering robust axonal regeneration at the lesion core. This approach achieves a regeneration level more than 30 times higher than that of naive treatment, facilitates spinal canal circuit reconstruction, and promotes motor function recovery after spinal cord injury in mice. Notably, in vitro studies have shown that human plasma exosomes (HP-exos) carrying RVG, ILP, and ISP peptides are safe and cause no liver or kidney toxicity when administered to nude spinal cord injury (SCI) mice [38]. These results suggest that targeting neurovascular dysfunction with EVs may offer a promising avenue to alleviate pain and improve structural integrity in IVDD. Taken together, these mechanisms illustrate the multifactorial nature of intervertebral disc degeneration and highlight potential engineering intervention targets, which are summarized in Table 1.

**Table 1:** Summary of engineered strategy for pathological mechanism. RVG: neuron-targeting peptide; VEGF: vascular endothelial growth factor; PlGF: placental growth factor; sFlt-1-Exo: sFlt-1-loaded exosomes

Pathological mechanism	Key targets	Engineering strategy	References
Inflammatory response	RASSF5, NLRP3, NF-κB	miR-532-5p, miR-302c, miR-217, lncRNA MALAT1	[16,19,20]
Oxidative stress	PI3K/Akt/mTOR, COL II, ACAN, Keap1-Nrf2, ATF6	PDA@Exo MN, SOD3, miR-141-3p, miR-31-5p	[25-28]
ECM dysregulation	COL2A1, SOX9, NF-κB, MMP3, MMP13	miR-214-3p, exoFGF2@ECM/Cu²⁺ hydrogel, CAP-Exo/siMMP13	[31-33]
Neurovascular abnormality	VEGF, PlGF, IL-6R, NFκB, TGF-β1/SMAD2/3	sFlt-1, miR-204, srIκB, Schwann cell, RVG, ILP, ISP	[34-38]

Hierarchical engineering strategies for EV modification

**Figure 3:**
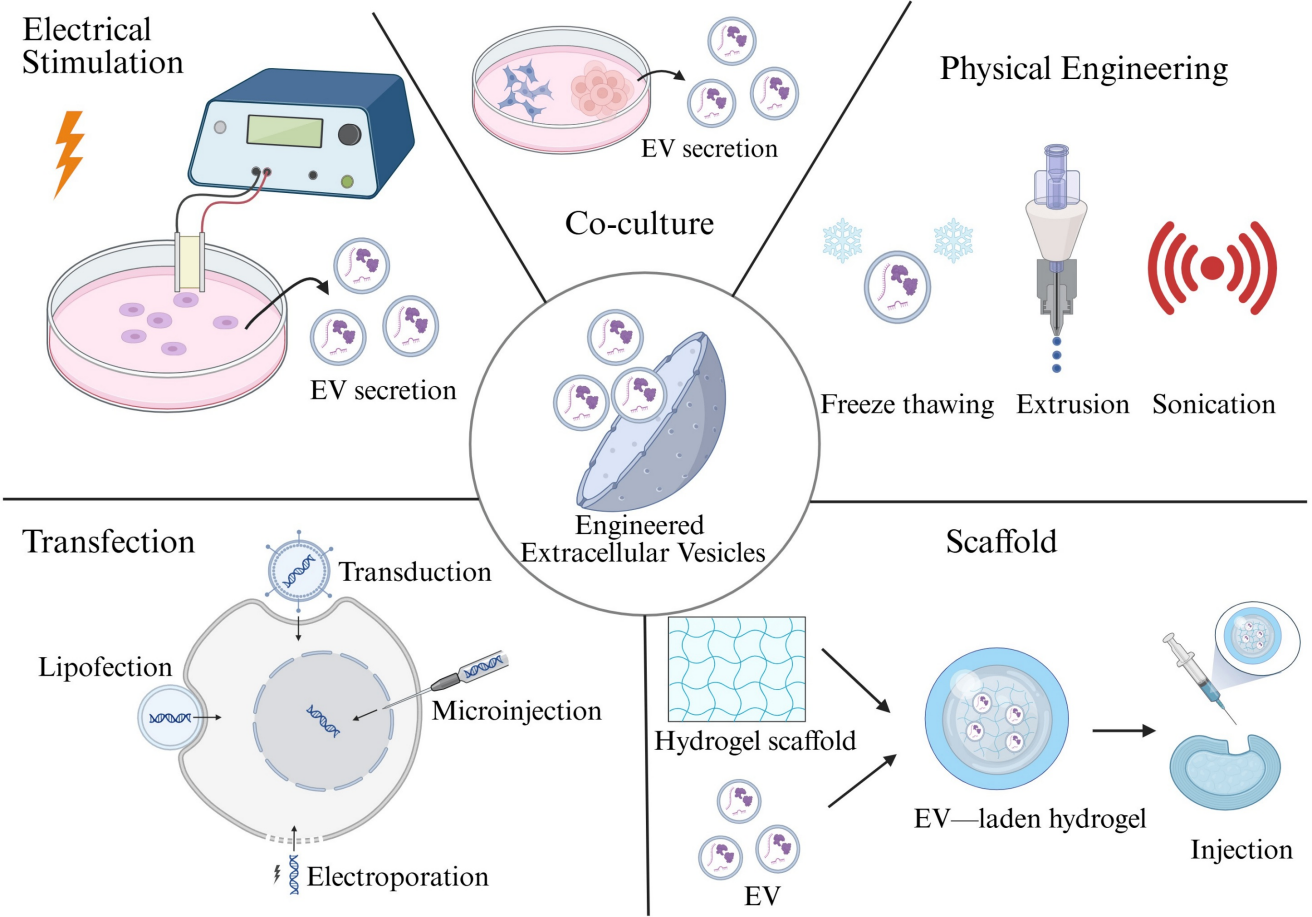
Engineering strategy for extracellular vesicles. This image is created by the authors of this study using BioRender.com (agreement number: FD28VP634G).

Engineered EVs represent an emerging therapeutic modality, wherein naturally secreted vesicles are modified through bioengineering approaches to enhance their functional attributes. These engineered vesicles can be equipped to deliver bioactive molecules, such as proteins, mRNA, microRNA, and therapeutic drugs, thereby augmenting intercellular communication and disease modulation [39,40]. Compared with native EVs, engineered EVs offer increased yield, improved targeting efficiency, enhanced therapeutic potency, and optimized drug delivery. Due to their intrinsic biocompatibility, low immunogenicity, and multifunctionality, they are considered ideal therapeutic carriers in regenerative medicine and precision therapies (Figure 3).

Electrical Stimulation-Mediated Engineering

Although nearly all tissue cells and body fluids can secrete EVs, the endogenous secretion level is often insufficient for clinical applications [41]. To overcome this limitation, external bioengineering stimuli, such as electrical stimulation, pharmacological agents, and electromagnetic waves, have been developed to upregulate EV production [39]. Electrical stimulation modulates cellular functions by delivering precise electrical signals that trigger intracellular signaling cascades and enhance endocytosis of exogenous molecules, thereby boosting EV secretion [42]. For example, in cardiac resynchronization therapy, electrical stimulation increases the expression of neutral sphingomyelinase 2, promoting EV release from cardiac mesenchymal stem cells and enhancing their cardioprotective capacity [43].

In a newly developed "cell nanoporation" technique, localized transient electrical stimulation increased exosome yield by up to 50-fold and enriched their mRNA content. This approach induces transient pore formation, enhanced multivesicular body biogenesis, membrane self-repair, and elevated calcium ion influx, thereby significantly enhancing vesicle functionality [44]. Additionally, EVs derived from human umbilical cord mesenchymal stem cells under microelectric field stimulation were found to carry lncRNA-MALAT1, which targets miR-22-3p to activate AMPK phosphorylation and Bcl-2 expression, ultimately inducing autophagy and reducing neuronal apoptosis [45].

Co-incubation With Bioactive Agents

Co-incubation is an in vitro bioengineering method that cultures multiple cell types together to facilitate intercellular communication, signal exchange, and microenvironmental modulation. For instance, EVs secreted by Talaromyces marneffei and co-incubated with THP-1 macrophages elevated IL-1β, IL-6, and IL-10 levels, and increased CD86 expression - indicative of M1 macrophage polarization [46]. Similarly, co-incubating neutral and cationic liposomes with tumor cells enhances exosome release in a dose-dependent manner. These exosomes exhibit distinct protein profiles depending on the liposome formulation, providing a novel platform for designing disease-specific EVs [47,48]. Furthermore, bone marrow MSC-derived exosomes co-incubated with H9C2 cardiomyoblasts elevated phosphorylated AKT levels and reduced apoptosis by delivering miR-144, which targets the PTEN/AKT signaling pathway [49]. Such cell interaction-based approaches offer versatile and scalable avenues to customize EVs for specific pathologies.

Genetic Engineering via Transfection

Transfection enables the introduction of exogenous nucleic acids into host cells for genetic modulation, allowing for cargo loading of EVs with therapeutic RNA or protein-encoding sequences. Techniques include physical (e.g., electroporation), chemical (e.g., liposome-mediated), and biological (e.g., viral vector) methods [50].

Engineered exosomes derived from M1 macrophages, transfected with NF-κBp50 siRNA and miR-511-3p, significantly enhanced M1 polarization. Moreover, surface modification with IL4RPep-1 peptide enabled targeting of IL-4 receptors on tumor-associated macrophages, inducing their reprogramming to the M1 phenotype and triggering potent antitumor immune responses in vivo [51]. Lentivirus-mediated transduction of L1210 leukemia cells produced exosomes (LEXs) enriched with CD80/CD86, which enhanced dendritic cell activation, promoted Th1 cytokine production, and induced leukemia-specific cytotoxic T lymphocyte responses. In vivo, LEXs delayed tumor progression and prolonged survival [52]. These findings illustrate the dual advantage of genetic loading and surface targeting in exosome engineering.

Physical Engineering Techniques

Physical engineering approaches, including freeze-thaw cycling, extrusion, and ultrasound, provide direct means to modify EVs post-isolation [53]. Repeated freeze-thaw cycles facilitate membrane fusion between liposomes and exosomes, allowing functional payload encapsulation [54]. The extrusion method passes drug-laden lipid mixtures through defined pore-size membranes under pressure, producing uniform, size-controlled vesicles with enhanced drug encapsulation and cellular uptake [55]. However, excessive pressure may compromise EV integrity [56].

Ultrasound applies acoustic forces that transiently permeabilize the EV membrane, enabling drug entry while preserving vesicle structure through membrane self-repair [57,58]. In a cancer model, Erastin (a ferroptosis inducer) and Rose Bengal (a photosensitizer) were loaded into EVs using ultrasound. Upon administration, this combined photodynamic strategy triggered tumor ferroptosis while minimizing systemic toxicity [59]. These physical strategies allow for precise control over drug loading and cargo functionality while maintaining structural fidelity.

EV-Scaffold Hybridization Strategies

Scaffold-based platforms enhance EV therapeutic efficacy by offering localized delivery, sustained release, and microenvironmental mimicry. Scaffold materials range from natural hydrogels to synthetic polymers and decellularized extracellular matrix (dECM) [60]. In a spinal cord injury (SCI) model, hUC-MSC-derived small EVs loaded with berberine were embedded in a GelMA hydrogel scaffold. This construct effectively modulated inflammation, reduced fibrosis, and promoted neural regeneration and motor recovery in rats [61]. In bone tissue engineering, poly (lactic-co-glycolic acid) (PLGA)/magnesium-gallic acid metal-organic framework (Mg-GA MOF) composite scaffolds anchored with human adipose-derived mesenchymal stem cell (hADSC)-derived exosomes induced osteogenesis and angiogenesis, and attenuated inflammation in vivo [62]. Scaffolds provide not only mechanical support but also an immune-modulatory interface, facilitating tissue integration [63].

Advancements in 3D bioprinting enable spatially precise EV placement. A dual-network hydrogel scaffold combining tissue-specific dECM and MSC-derived exosomes guided chondro-osteogenic differentiation and promoted cartilage-bone regeneration in vivo [64]. This biofabrication strategy represents a breakthrough in cell-free regenerative medicine.

In summary, hierarchical engineering strategies, including electrical stimulation, co-incubation, transfection, physical manipulation, and scaffold-assisted delivery, have revolutionized EV-based therapies. Despite these advances, challenges remain in standardization, targeting specificity, scalability, and long-term safety. The integration of single-cell omics and AI-guided design is expected to drive the development of next-generation programmable EVs, enabling personalized, precision therapies for intervertebral disc degeneration and beyond.

Targeted EV-based therapeutics for specific degenerative phenotypes

Hydrogel-Embedded EV Delivery Systems

Compared to native EVs, engineered EVs derived from MSCs genetically modified to overexpress Cavin-2 exhibit significantly enhanced endocytic efficiency in NPCs stimulated with TNF-α. In both three-dimensional hydrogel co-culture systems and ex vivo organ culture models, these EVs effectively attenuated NPC apoptosis and delayed the pathological progression of IVDD [65]. To address degenerative mechanisms, such as NPC senescence and ECM degradation, Liu et al. identified glutaredoxin 3 (GLRX3) as a critical redox regulator associated with NPC aging and disc degeneration [66]. They engineered MSC-derived EVs enriched with GLRX3 (EVs-GLRX3) and incorporated them into a supramolecular hydrogel composed of disc tissue-mimicking biopolymers. These EVs enhanced the antioxidant defense of NPCs, prevented the accumulation of ROS, and suppressed cellular senescence in vitro. In a rat IVDD model, GLRX3-loaded hydrogels mitigated mitochondrial dysfunction, reduced NPC senescence, promoted ECM synthesis, and ameliorated disc degeneration by restoring redox homeostasis.

MicroRNA-Enriched EV Therapies

MicroRNAs (miRNAs) are endogenous, small non-coding RNAs that regulate gene expression by promoting mRNA degradation or inhibiting translation. In IVDD, miRNAs influence key cellular processes including apoptosis, ECM homeostasis, inflammation, oxidative stress, and angiogenesis.

Zhang et al. reported that aged NPCs secrete EVs containing miR-27a-3p, which polarize macrophages toward the pro-inflammatory M1 phenotype via activation of the cGAS/STING pathway, exacerbating NPC senescence and degeneration [67]. Engineered EVs carrying overexpression plasmids for ataxia-telangiectasia and Rad3-related protein (ATR) reduced DNA damage-induced NPC senescence and inhibited the cGAS/STING pathway, effectively attenuating IVDD progression.

Jiang et al. developed a polymeric nanoparticle system modified with NPC-specific aptamers for the targeted delivery of miR-150-5p inhibitors [68]. This system reduced NPC senescence in vitro and exhibited sustained therapeutic efficacy for over three months in vivo. The NPC microenvironment facilitated lysosome-evading delivery, significantly reducing the secretion of senescence-associated secretory phenotype (SASP) factors. Mechanistically, miR-150-5p inhibition downregulated the NF-κB pathway by targeting FBXW11 and suppressing TAK1 ubiquitination, thus slowing IVDD progression in an acupuncture-induced mouse model.

Bioengineered Scaffolds for EV Retention and Release

The integration of EVs with bioengineered scaffolds presents a powerful strategy for IVDD therapy. Scaffolds replicate the native intervertebral disc microenvironment, provide structural support, and enable localized, sustained delivery of therapeutic EVs [69].

For instance, cartilage endplate stem cells engineered to overexpress sphingosine kinase 2 (Sphk2) were embedded in rib cartilage-derived ECM hydrogels, producing Sphk2-rich EVs. These EVs penetrated the annulus fibrosus and activated the PI3K/AKT pathway in NPCs, thereby promoting autophagy and suppressing SASP protein expression, ultimately delaying disc degeneration [70]. Moreover, Peng et al. developed an RGD-functionalized nucleus pulposus matrix hydrogel (RGD-DNP) that exploits RGD-integrin interactions on EV membranes [71]. This hydrogel enhanced sEV retention in vitro and in vivo, promoted NPSC migration, reduced senescence markers (p16, p21, p53), and restored cell cycle progression. Mechanistically, sEVs delivered miR-3594-5p to inhibit the HIPK2/p53 axis, attenuating NPSC senescence. Notably, the therapeutic effect of low-dose sEVs delivered via RGD-DNP hydrogel was equivalent to that of a high-dose free sEV injection.

To address the complex structure of the annulus fibrosus (AF) and its limited regenerative capacity, a core-shell nanofiber scaffold was fabricated via electrospinning. The scaffold co-delivered transforming growth factor-β3 (TGF-β3) and ibuprofen (IBU) in a biphasic release profile - rapid anti-inflammatory IBU release followed by sustained TGF-β3 delivery to promote ECM synthesis. Implanted into a box-shaped AF defect model, this structure effectively preserved disc biomechanics, inhibited inflammation, and promoted ECM regeneration across multiple in vivo studies [72]. These findings highlight the versatility of scaffold-EV platforms for delivering targeted, long-lasting treatments in disc regeneration and clinical translation.

Smart Composite Platforms Integrating EVs and Responsive Scaffolds

Emerging technologies have introduced smart delivery systems that combine mechanical energy harvesting, optogenetics, and bioengineering for precise EV-based therapies in IVDD. Zhang et al. designed a self-powered platform that utilizes triboelectric pulses from wearable devices, coupled with optogenetic stimulation, to trigger the targeted release of EVs carrying TRAM1 protein into senescent NPCs [73]. This restored TREX1-mediated DNA clearance, reduced inflammation, and significantly alleviated disc degeneration in animal models.

Hu et al. developed a linear microneedle system (T-MN) tailored to the ring-shaped structure of the AF [74]. Fabricated via 3D printing using silk fibroin methacrylate (SilMA) and laminin composites, this platform was loaded with bone marrow MSC-derived exosomes and miR-378 - a key regulator of mitochondrial autophagy. The T-MN@EXO@miR-378 system promoted mitochondrial homeostasis, AF cell migration, and proliferation, while preventing pathological ECM remodeling and effectively arresting IVDD progression.

**Figure 4:**
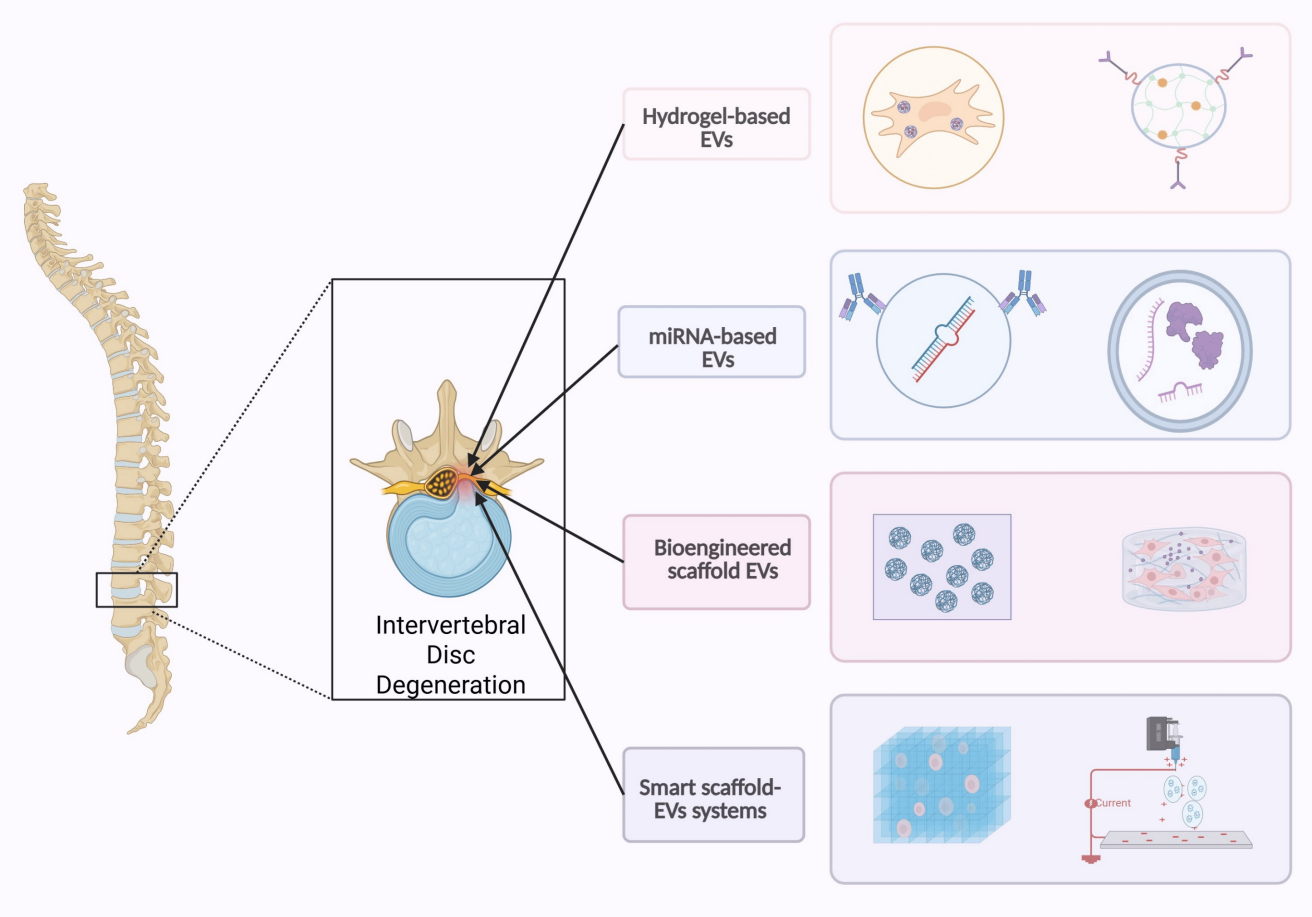
Precise treatment of intervertebral disc degeneration with different types of engineered EVs. This image is created by the authors of this study using BioRender.com (agreement number: ON28VQJXHM). EV: extracellular vesicle

Together, these advanced delivery platforms illustrate the promise of intelligent, stimulus-responsive therapies that synergize engineered EVs and precision biomaterials, offering transformative strategies for degenerative disc disease treatment (Table 2, Figure 4).

**Table 2:** Summary of engineered EV-based therapeutic strategies for IVDD. IVDD: intervertebral disc degeneration; EV: extracellular vesicle; NPC: nucleus pulposus cells; NP: nucleus pulposus; ROS: reactive oxygen species; GLRX3: glutaredoxin 3; ECM: extracellular matrix; ATR: ataxia-telangiectasia and Rad3-related protein; SASP: senescence-associated secretory phenotype; IBU: ibuprofen

Category	Engineering strategy	Core mechanism	Experimental outcomes	References
Hydrogel-based EVs	Cavin-2 gene-edited mesenchymal stem cells.	Enhanced EV endocytosis.	Reduced NP cell apoptosis.	[65]
	GLRX3-enriched EVs combined with supramolecular hydrogel.	ROS clearance, prevention of senescence amplification.	Promoted ECM deposition, alleviated NP degeneration.	[66]
miRNA-based EVs	ATR overexpression plasmid.	Inhibition of cGAS/STING pathway.	Alleviated NPC senescence and IVD pathology.	[67]
	Aptamer-modified nanoparticles delivering miR-150-5p inhibitor.	Suppression of FBXW11/TAK1 ubiquitination.	Delayed NPC degeneration.	[68]
Bioengineered scaffold EVs	Lenti-Sphk2 exosomes combined with ECM-Gels hydrogel.	Activation of PI3K/AKT pathway, SASP inhibition.	Inhibited NPC senescence.	[70]
	RGD-DNP composite hydrogel loaded with sEVs.	miR-3594-5p targeting HIPK2/p53 pathway.	Enhanced NPC migration, mitigated senescence.	[71]
	Electrospun core-shell scaffold loaded with TGF-β3/IBU.	TGF-β3 promotes ECM synthesis, and IBU reduces inflammation.	Biomimetic AF structure, maintained IVD biomechanics.	[72]
Smart scaffold-EVs systems	Triboelectric-responsive microneedles with TRAM1-EVs.	Restored TREX1 clearance in NPCs.	Suppressed inflammation, slowed IVDD progression.	[73]
	T-MN@EXO@miR-378 microneedles (3D-printed SilMA scaffold).	miR-378 regulates mitophagy and inhibits pathological ECM remodeling.	Promoted AF repair, effectively halted IVDD progression.	[74]

Current challenges 

Extracellular vesicles serve as natural mediators of intercellular communication by transferring proteins, nucleic acids, and metabolites between cells. They exhibit unique therapeutic potential for intervertebral disc degeneration through the regulation of inflammation, extracellular matrix restoration, and cytoprotection. Their intrinsic low immunogenicity, strong biocompatibility, and capacity for long-range molecular transfer make them promising candidates for cell-free regenerative therapy [75]. However, the clinical translation of EV-based therapies faces several significant challenges discussed further.

Large-Scale Production and Quality Control

Reproducible and standardized EV production remains a primary challenge. Achieving batch-to-batch consistency in purity, yield, and stability is crucial for therapeutic reproducibility. Moreover, senescent NPCs demonstrate reduced EV endocytosis, compromising efficacy. Surface modification, such as ligand conjugation or protein engineering, can enhance uptake efficiency and restore cellular responsiveness [65].

Targeting Specificity and In Vivo Biodistribution

Although EVs exhibit favorable biocompatibility, their in vivo targeting and homing efficiency remain suboptimal. Genetic or chemical engineering of EV surface proteins can improve site-specific delivery [76]. However, such modifications must preserve vesicle integrity and function, necessitating sophisticated validation protocols.

Cargo Loading Capacity and Functional Preservation

EVs have limited intrinsic cargo-loading capacity, restricting their application in complex therapies. Emerging approaches, such as vesicle membrane remodeling, fusion with synthetic nanocarriers, or the use of biomimetic scaffolds, can expand this capacity [77]. However, increasing loading efficiency while maintaining vesicle stability and bioactivity remains technically demanding.

Clinical Translation and Regulatory Oversight

Despite encouraging preclinical findings, clinical adoption requires rigorous evaluation of safety, efficacy, and dosage. Scalable purification and delivery platforms must comply with good manufacturing practices. Furthermore, regulatory frameworks addressing immunogenicity and long-term safety, particularly for allogeneic or xenogeneic EVs, are essential for successful clinical transition [78].

Future directions and technological integration

To overcome current barriers, interdisciplinary strategies are reshaping the landscape of EV-based therapeutics. Engineered EVs, optimized through bioengineering and molecular design, are at the forefront of next-generation regenerative interventions.

Engineered EVs and Precision Modulation

Tailored EVs incorporating surface ligands, enriched cargos, or modified biogenesis pathways have shown enhanced targeting precision, bioavailability, and therapeutic efficacy. These innovations hold potential not only for IVDD but also for other degenerative and inflammatory diseases [79].

Integration of CRISPR/Cas9 and EV Platforms

The CRISPR-Cas9 gene editing system presents a powerful tool for targeted therapeutic applications using EVs. Engineered EVs can encapsulate Cas9-sgRNA complexes, often through lentiviral transduction of donor cells, to enable precise gene editing in recipient cells [80]. Despite this promise, several translational challenges remain, including off-target editing, immunogenicity, and delivery efficiency. To mitigate these risks, researchers are developing high-fidelity Cas9 variants, such as HypaCas9, and exploring surface modifications (e.g., polyethylene glycol conjugation) to enhance EV stability and specificity [81,82]. These strategies aim to improve editing precision while reducing potential adverse effects, laying a foundation for safer clinical applications.

Ethical and Biosafety Considerations

The development of engineered or gene-edited EVs raises important biosafety and ethical concerns. Potential risks include oncogenic cargo transfer and unintended genomic alterations. Thorough safety assessments, covering tumorigenicity, telomerase activity, and long-term genotoxicity, are necessary. Ethical governance and transparent regulatory oversight are essential to ensure responsible clinical translation.

Interdisciplinary and Computational Synergies

Bioresponsive scaffolds and hydrogel-based carriers can protect EVs and enhance their retention at target sites [83]. Advances in synthetic biology, biomaterials, and computational modeling are converging to accelerate EV innovation [84,85]. At the same time, artificial intelligence (AI)-driven predictive modeling of EV-receptor interactions can guide rational vesicle design and personalized therapy development [86].

## Conclusions

Engineered extracellular vesicles represent a transformative paradigm in regenerative medicine for intervertebral disc degeneration. By enabling targeted, biocompatible, and cell-free therapeutic delivery, they address several limitations inherent to conventional cell-based approaches. Although substantial progress has been made, challenges related to standardization, scalability, and safety validation must still be addressed before clinical application can be realized.

Future success will depend on collaborative efforts that unite molecular biology, materials science, bioengineering, and regulatory science. Through such integrated innovation and ethical stewardship, engineered EVs may soon transition from experimental platforms to clinically viable therapeutics, offering renewed potential for restoring disc health and combating degenerative spinal diseases.
